# The generic Informed Consent Service gICS^®^: implementation and benefits of a modular consent software tool to master the challenge of electronic consent management in research

**DOI:** 10.1186/s12967-020-02457-y

**Published:** 2020-07-29

**Authors:** Henriette Rau, Lars Geidel, Martin Bialke, Arne Blumentritt, Martin Langanke, Wenke Liedtke, Sandra Pasewald, Dana Stahl, Thomas Bahls, Christian Maier, Hans-Ulrich Prokosch, Wolfgang Hoffmann

**Affiliations:** 1 Trusted Third Party of the University Medicine Greifswald, Ellernholzstr. 1-2, 17475 Greifswald, Germany; 2 Institute for Community Medicine Section Epidemiology of Health Care and Community Health, University Medicine Greifswald, Ellernholzstr. 1-2, 17475 Greifswald, Germany; 3 Protestant University of Applied Sciences in Bochum, Immanuel-Kant-Str. 18-20, 44803 Bochum, Germany; 4 Faculty of Theology, University of Greifswald, Am Rubenowplatz 2-3, 17487 Greifswald, Germany; 5 Chair of Medical Informatics, Friedrich-Alexander-Universität Erlangen-Nürnberg, Wetterkreuz 13, 91058 Erlangen, Germany

**Keywords:** Consent management, GDPR, General data protection regulation, Informed consent

## Abstract

**Background:**

Defining and protecting participants’ rights is the aim of several ethical codices and legal regulations. According to these regulations, the Informed Consent (IC) is an inevitable element of research with human subjects. In the era of “big data medicine”, aspects of IC become even more relevant since research becomes more complex rendering compliance with legal and ethical regulations increasingly difficult.

**Methods:**

Based on literature research and practical experiences gathered by the Institute for Community Medicine (ICM), University Medicine Greifswald, requirements for digital consent management systems were identified.

**Results:**

To address the requirements, the free-of-charge, open-source software “generic Informed Consent Service” (gICS^®^) was developed by ICM to provide a tool to facilitate and enhance usage of digital ICs for the international research community covering various scenarios. gICS facilitates IC management based on IC modularisation and supports various workflows within research, including (1) electronic depiction of paper-based consents and (2) fully electronic consents. Numerous projects applied gICS and documented over 336,000 ICs and 2400 withdrawals since 2014.

**Discussion:**

Since the consent’s content is a prerequisite for securing participants’ rights, application of gICS is no guarantee for legal compliance. However, gICS supports fine-granular consents and accommodation of differentiated consent states, which can be directly exchanged between systems, allowing automated data processing.

**Conclusion:**

gICS simplifies and supports sustained IC management as a major key to successfully conduct studies and build trust in research with human subjects. Therefore, interested researchers are invited to use gICS and provide feedback for further improvements.

## Background

Research becomes more and more complex, increasingly involving (inter-)national partners collecting, transferring, processing and storing growing amounts of data from different sources. Consequently, complying with relevant (legal and ethical) regulations is often a challenge. The requirement that each participant in a research project provides an “informed consent” (IC) is widely seen as a cornerstone of the ethical acceptability of medical research involving human subjects.

The concept of informed consent is often referred to as the ethical and legal answer of the Nuremberg Court [1] to the horrific experiments of Nazi doctors with hospital patients or inmates of the concentration camps. However, this concept has several origins.

In the U.S., the Tuskegee Syphilis Study was conducted between 1932 and 1972. In this study, participants received false information about the nature and the duration of the research, which included withholding available treatment from affected patients to observe the “natural course of the disease” – with numerous severe consequences for their own and their relatives’ lives [2, 3]. The example of this study demonstrates that massive IC-related shortcomings endangering the health of the participants and violating their basic human rights occurred not only in interventional research but also in pure observational epidemiological research. The immense mistreatment of participants within the Tuskegee study led in great parts to the Belmont Report – one of the most influential research ethics codices until today [4].

Both lines, the extraordinary abuse of people by Nazi doctors as well as the less known mistreatment of study participants within mainstream medical science, triggered a development after the end of the 2nd World War that resulted in the publication of several research ethics codices, defining and protecting participants’ rights in the sphere of human subject research. Besides the well-known Declaration of Helsinki that appeared in its first version in 1964 [5] numerous codices and laws today regulate the interaction and treatment of people participating in medical research. All pertinent regulations emphasise the IC as an inevitable element of the process of research with human subjects. These days the focus on direct physical and psychological violations of study participants and their relatives extends to harm resulting from the potential misuse of participants’ data. In the era of international multi-site studies and “big data medicine” these aspects of IC become more and more relevant. Today, handling patient data for research needs to be compliant with legal requirements stated by the EU General Data Protection Regulation (GDPR) as well as national legislation, e.g. the German Data Protection Act (German: Bundesdatenschutzgesetz, BDSG) and the Data Protection Act of the respective federal state (German: Landesdatenschutzgesetze, LDSG).

According to MITRE [6] “[…] consent management is a system, process, or set of policies that enables […]” participants to decide what healthcare providers and researchers are allowed to do with their health information, and to extend this to all kinds of specific personal information. Patient’s health information, i.e. medical data, are classified as personal data, which are considered particularly sensitive, and, thus, can only be processed with a patient’s informed consent [7]. Deviating from MITRE [6], this paper uses the term “informed consent” for a participant’s written decision defining the use of personal health information for research purposes only (as opposed to use for treatment, reimbursement or other purposes).

Informed consent means that individuals are not only informed about the contents of a study but also understand the information given. Thus, the consent should be discussed with the eligible participant and only signed *after* this discussion but *before* the data collection [8].

Usually, the consent of an eligible study participant is captured on a paper-based form that is signed by the participant. Such a paper-based approach usually does not include structured information and is not machine-readable. An electronic consent management facilitates the capturing of consent in a digital format—either digitising a paper-based consent (including scans of consents) or directly capturing the consent digitally via electronic consent mechanisms. Consequently, the participant’s permissions for usage together with his/her choice of restrictions including partial and complete withdrawals can be handled based on automated algorithms by an Informed Consent Management System [6]. Electronic consent management has major advantages compared to conventional manual handling, which leads to repeated searching for a participant’s specific written statement, uncontrolled storage of paper-based forms, and availability at only one location. Additionally to facilitating processes—especially regarding modular consents—and reducing time demands, electronic consent management has logistic advantages that can be substantial. For example, the German National Cohort (NAKO, [9]) stores more than 307,000 consents (as of October 2019), which corresponds to paper-based forms in excess of no less than 10 tons.

Despite these advantages, electronic consent mechanisms were still an unsolved issue in 2007 [8], and even in 2014, MITRE [6] stated that “[…] electronic consent management is not yet common practice […]” and it was still common to collect consents solely on a paper form.

A literature search regarding “Informed Consent Management” showed that existing (open-source) software tools for consent management differ in their use cases and application context. For example, the consent wizard of the Technology, Methods, and Infrastructure for Networked Medical Research e. V. (TMF) [10] supports its users in the creation of consent documents, but does not offer help in managing, versioning, modularising or querying consents and withdrawals digitally. The Consent Management Suite (COMS) [11] facilitates the creation and administration of consent documents in the context of medical treatment. However, it also does not support versioning, modularising or querying consents and withdrawals digitally. The School of IT and Computer Science at the University of Wollongong, Australia, developed the eMedical Book Consent providing a “General Consent with specific Denials” or a “General Denial with specific consents” prototypes, respectively, for electronic health records (EHR) [8]. In this case, the consent is a security profile, granting or denying access to, amongst others, the patient’s medical history, examination, consultation and health assessment [8]. It may also be useful for research, but the software solution seems not to be accessible to other research facilities. Additionally, commercial products exist [12, 13]. However, those tools are too expensive for most research projects with limited resources.

Consequently, there is a need for computer-assisted IC processes with sufficient flexibility of applications in a large variety of settings and use cases. The University Medicine Greifswald developed a scalable generic– thus, portable and adaptable - approach to implement a generic Informed Consent Service (gICS) to facilitate consent management for all possible study types and settings, with a focus on epidemiological research studies and registries. gICS was published as free-of-charge and open-source software within the MOSAIC project [14] (funded by the German Research Foundation (*HO 1937/2*-*1*)).

This paper discusses the organisational development and technical implementation of the software solution gICS for the creation, management and modularisation of informed consents as well as its support of policy-specific automated queries and withdrawals. Additionally, it evaluates the benefits for research projects using this modular consent software tool.

## Methods

### Requirements regarding electronic IC management

An electronic IC needs to cover the same requirements as a completed and signed paper-based consent (cf. [15]). Based on Bahls et al. [15], MITRE [6], the TMF guideline on data protection [16], Schreiweis et al. [17], and practical experience with research projects conducted by the Institute for Community Medicine (ICM) Greifswald, a consent management system should initially fulfil the following requirements for digitally recorded ICs (see Table 1).

**Table 1:** List of requirements for a comprehensive informed consent management. Based on Bahls et al. [15] and modified with [6], [16] and [17]

No.	Requirement and/or use case
1	Support of a general consent form for a research project, e. g. a digital consent template
2	Support of individual participant consents to digitally store filled-in participants’ consents
3	Clarity and transparency regarding each consent status to support Use and Access processes in compliance with data protection regulations
4	Editing and updating, i.e. consent templates, and enabling the participant to change his/her will any time
5	Support of consent exclusions [18], e.g. the participant can actively exclude the collection, use or processing of personal health information and/or biological samples or limit them to certain types of research
6	Possibility to define any number of (external) properties to support study-specific requirements
7	Possibility to define free text fields to support study-specific input fields, e. g. study site or specific dates/timestamps
8	Possibility to withdraw consent (fully or partially) in compliance with participants’ right to withdraw and to be forgotten (Art. 7 and 17 GDPR)
9	Support of consent versioning to support multiple consent versions within a study, e. g. to track changes over time or to provide consents in multiple languages
10	Possibility to freely configure automatable queries for consent status, e. g. for Use and Access processes
11	Possibility to define policies and combine them into modules to support fine-granular depiction of the participant’s expressed consent
12	Possibility to define mandatory policies/modules
13	Possibility of automated search or query of individual consented consent forms, policies, modules or specific identifiers, e. g. case number, to support use cases for data trustees such as List all participants, who consented to a specific policy List all consent forms existing for a study List all digital consents of a study, for which no scan of the paper-based IC is attached List all policies to which a participant has consented to List all consent forms, which exist for a participant Display the current consent form existing for a participant Answer the query, whether a given participant consented to a specific policy
14	Support of exporting consented cases, e. g. by providing a list of participants’ pseudonyms with valid consents
15	Integration of paper-based workflows, e. g. attaching documents to a participant’s digital consent
16	Management of domains (e. g. multiple projects, different study sites, or countries)
17	Intuitive usage and support of use cases, e. g. using an frontend with menu items like “search”
18	Possibility to define the time of validity of a consent

According to the literature search conducted regarding State of the art [8, 10, 11], existing tools for consent management are not always available or affordable to the scientific community or do not cover most of the stated requirements (see Table 1) regarding IC management.

### Concepts of gICS

#### Modular approach of consent management with gICS

**Fig. 1:**
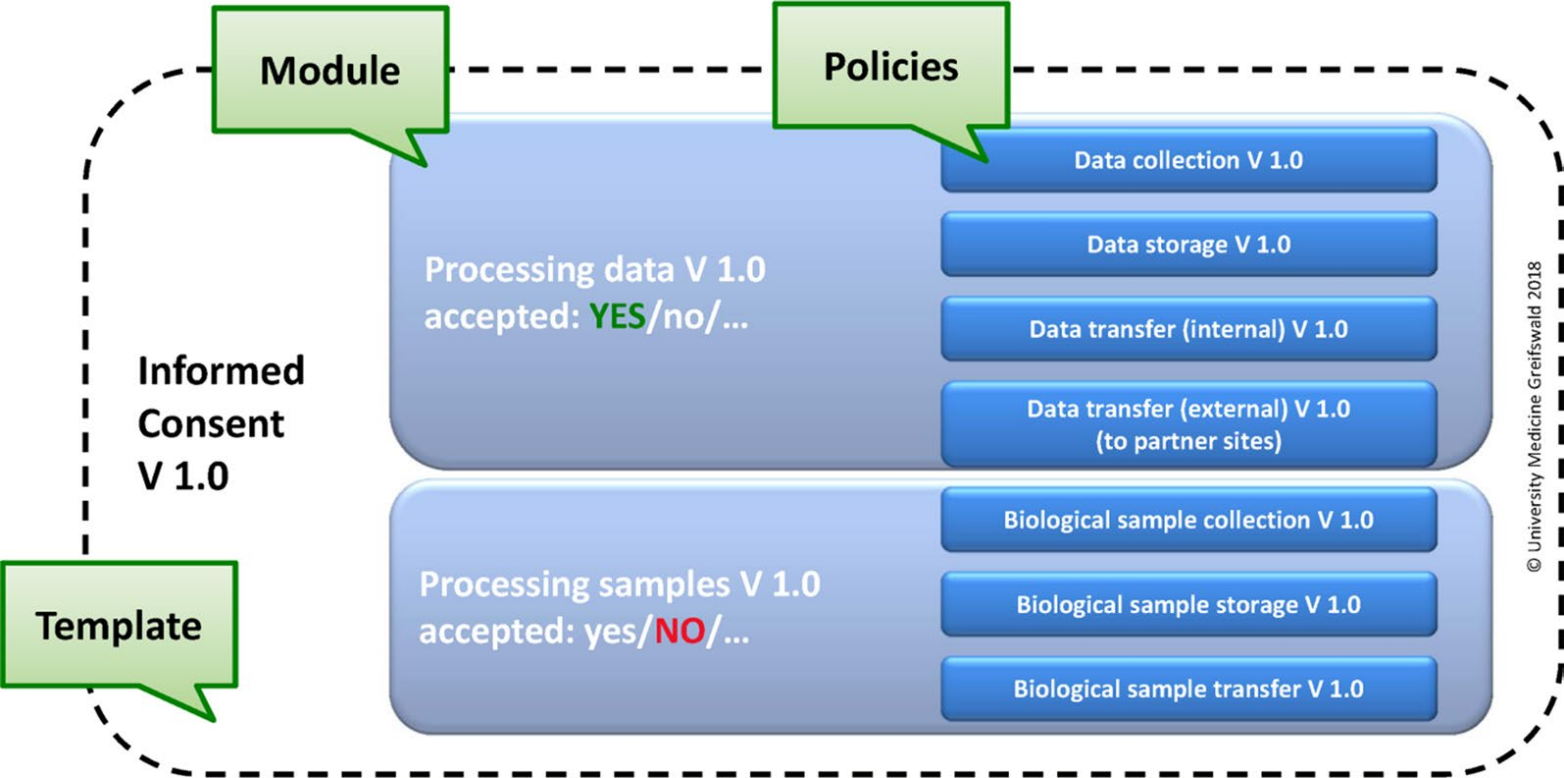
Structure of a modular Informed Consent within gICS

A valid informed consent needs to reflect a participant’s willingness in sufficient detail. To ensure the respective granularity, consents within gICS are modular based on policies (see Fig. 1). A policy represents a decision or stated will, e. g. to allow data collection, or storage of biomaterials. That way the study participant can permit data collection but prohibit long time data storage or agree to biomaterial sampling but exclude, for example, DNA-based analyses. A process can address different policies; hence, aggregating related and logically coherent policies into modules can be appropriate. For example, a module “processing research data” combines and encapsulates policies for collecting, transferring (internally) and storing health information and descriptive formatted text. Only if all policies of this module are consented, data collection is worthwhile. Therefore, participants are enabled to consent to multiple policies at the same time. Additionally, such a module can also be flagged as “mandatory”, e. g. as a necessary condition for study participation.

Each documented IC is based on a versioned template. A template determines the content and structure of the consent form including an introductory text (header), selectable modules (e. g. accepted, declined, withdrawn) and a closure text (footer) as well as complementary information, e.g. order of modules, definition of obligatory modules as well as free text fields. Every template consists of at least one module but can accommodate any number of modules. For example, within a module the study participant is asked whether or not he wants to be informed about results and incidental findings from diagnostic procedures. Another module could ask the study participant for authorisation to contact third parties, e. g. his/her general practitioner, health insurance company or professional/family care giver.

Modules and policies can be freely combined, allowing for flexible and individualised usage for a large variety of studies. However, each policy must only occur once a template. This requirement results from the necessity to be consistent throughout the informed consent at any point in time. This approach eradicates the possibility of contradictory consent states: If a policy appears in different modules within the same consent template, a participant might first decline this policy in the first module and, afterwards, consent to the same policy in the second module leading to inconsistencies.

Policies, modules and templates are always version-specific. This means that each change results in a new version of a study’s consent. Therefore, subsequent changes of contents and wording in already consented policies, modules and templates lead automatically to a new version, respectively. Therefore, contents and wording of already consented parts remain untouched.

Contextual domains are used as organisational unit to manage policies, modules and templates. Domains can be projects, study sites, or institutions and support the provision of context-related data, e. g. logos or versioning details.

#### Possible consent states and limitations

After introducing the concept of policies and modules, consent states are used to depict the participant’s will. Therefore, documentation should allow different status values.

Based on research studies, the following states are used:

- Accepted—the participant answered with “yes” to a policy, e. g. “Do you consent to data capture for this research project?”
- Declined—the participant answered with “no” to a policy, e. g. “Do you consent to data storage for this research project?”
- Unknown—the policy did not apply or the participant could not choose between any answer options for this policy. For example, this applies to cases, where a policy was added later to an IC template leading to a new version. Participants with older IC template versions had not been asked for this policy, and consequently the status is set to “unknown”.
  - Not_asked—specifying “unknown”: this policy did not apply and the participant wasn’t asked (e.g. female-specific issues in male participants)
  - Not_Chosen—specifying “unknown”: the participant didn’t answer the question at all
- Withdrawn—the policy was withdrawn by the participant
- Invalidated—the participant was excluded from the research project retrospectively, or data capture was invalidated for formal or technical reasons
- Refused—the participant was asked but refused to answer, e. g. “Do you want to participate in a research project?” during patient admission at a hospital
- Expired—the policy expired, e. g. a child’s consent was filed but now the person is of full age.

Furthermore, a participant’s consent can be subject to limitations regarding its period of validity. This can be either a fixed date, e. g. end of the research project, or a dynamic date. For example, if consent was given for a child by a custodial parent, it will only be valid until the child is of full age (e. g. 18^th^ birthday in Germany) and/ or has full legal capacity. It will expire as soon as the young participant is legally permitted to decide on his/her own and, consequently, has to sign a new consent.

## Results

Resulting from the lack of a software tool addressing all requirements as listed in Table 1, the generic Informed Consent Service gICS was provided within the MOSAIC project to manage consents for a variety of scientific use cases, especially within research studies. The gICS addresses all requirements listed in Table 1. gICS (current version: 2.10.0), was developed by the Institute for Community Medicine Greifswald and is used as a central tool by the Independent Trusted Third Party (TTP) [19] of the University Medicine Greifswald for research since 2014. It aims to fulfil all important requirements concerning IC management including assisting the two possible consent models—opt-out-model (implied consent) and opt-in-model (express consent)—by deposing a digital consent for each participant and updating it for any changes over the full life cycle of the study data.

### Implementation**—**architecture, distribution and web interface

gICS is an open source tool, licensed under AGPLv3 as part of the MOSAIC-project [14]. It is free of charge and available from the official homepage of the Trusted Third Party of the University Medicine Greifswald [20], GitHub [21] and the TMF ToolPool [22].

**Fig. 2:**
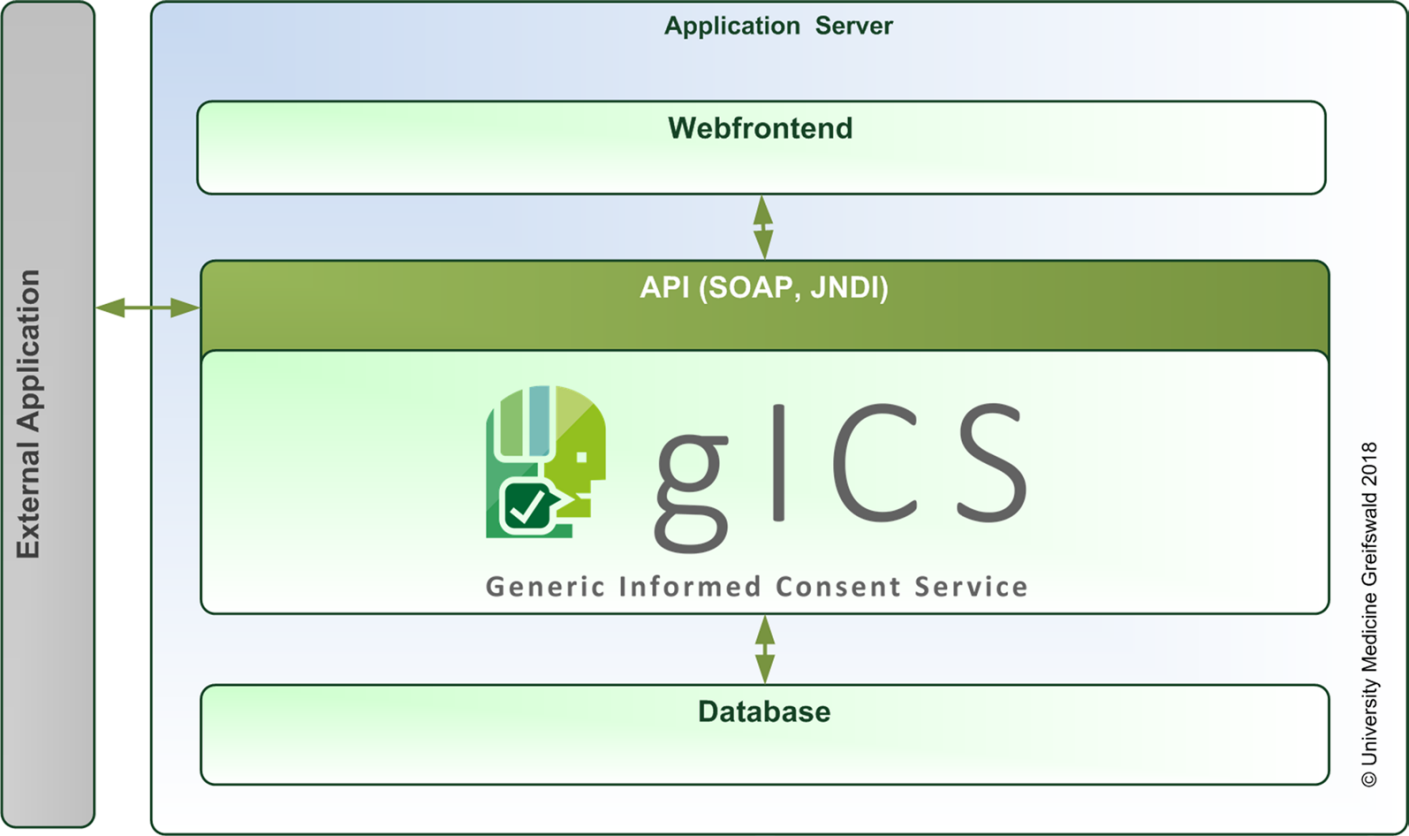
Architecture of the gICS application

gICS was developed as a 3-layer-architecture (Java EE) with a standardised web service-interface by using SOAP (see Fig. 2), optimised for MySQL.

As depicted in Fig. 2, gICS is a server application accessible via the provided web-services and/or the web-based user interface. The service-oriented architecture (SOA) allows the centralised provision of necessary services and their use via a uniform web-based user interface. All accesses to databases, the communication between client and server as well as external interfaces via SOAP and JNDI are coordinated by the necessary application server.

**Fig. 3:**
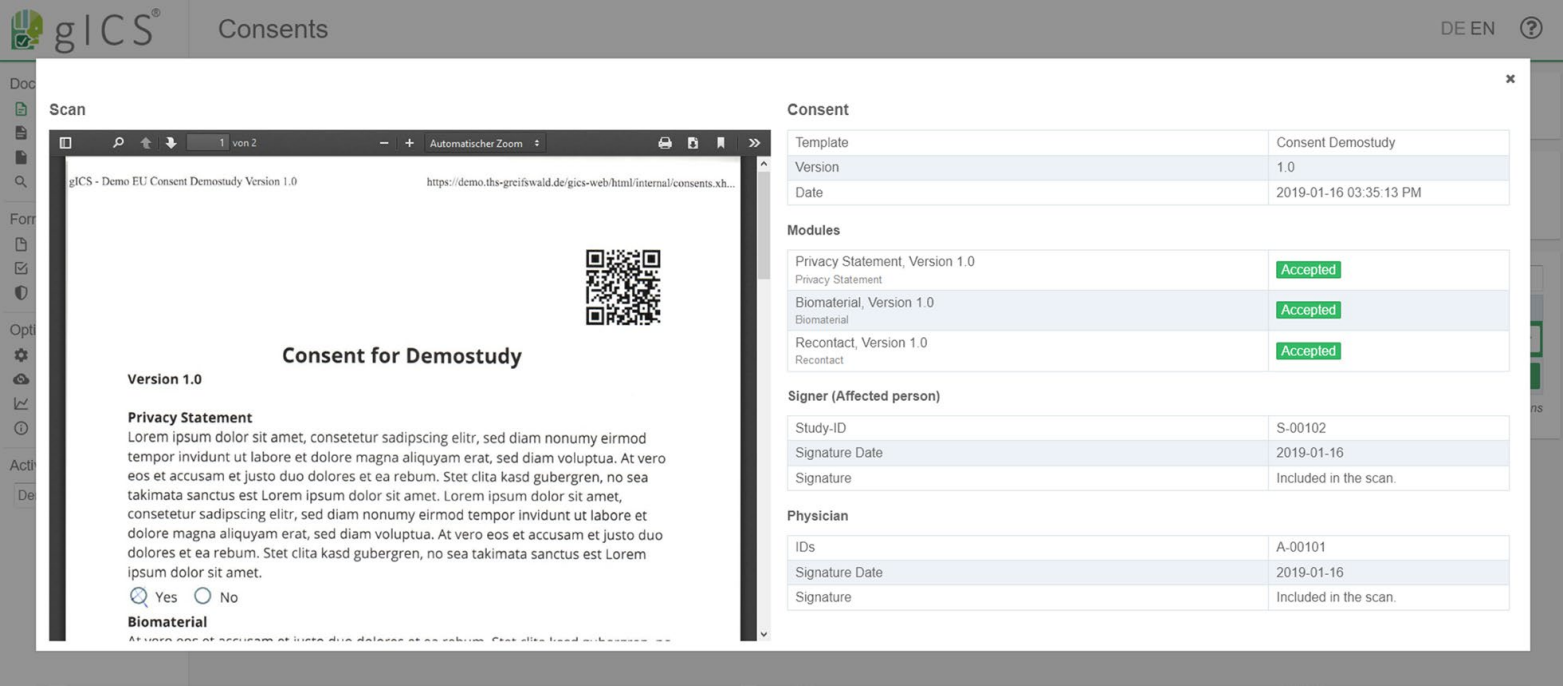
Web-based user interface of the gICS application

Using the web-based user interface (see Fig. 3) to work with gICS allows researchers to use the application as needed, guaranteeing an easy way to create, edit and monitor policies, modules and consent templates. gICS’ SOAP interface (gicsService, v.2.9.1) provides functionalities regarding consent management, queries, checks and validation, workflows and support functions. For example, policy-specific queries within gICS include listing all consents for a specific consent template, all consents for a specific domain without a scan of the paper-based consent or retrieving the current consent for a specific person.

### Usage of gICS

**Fig. 4:**
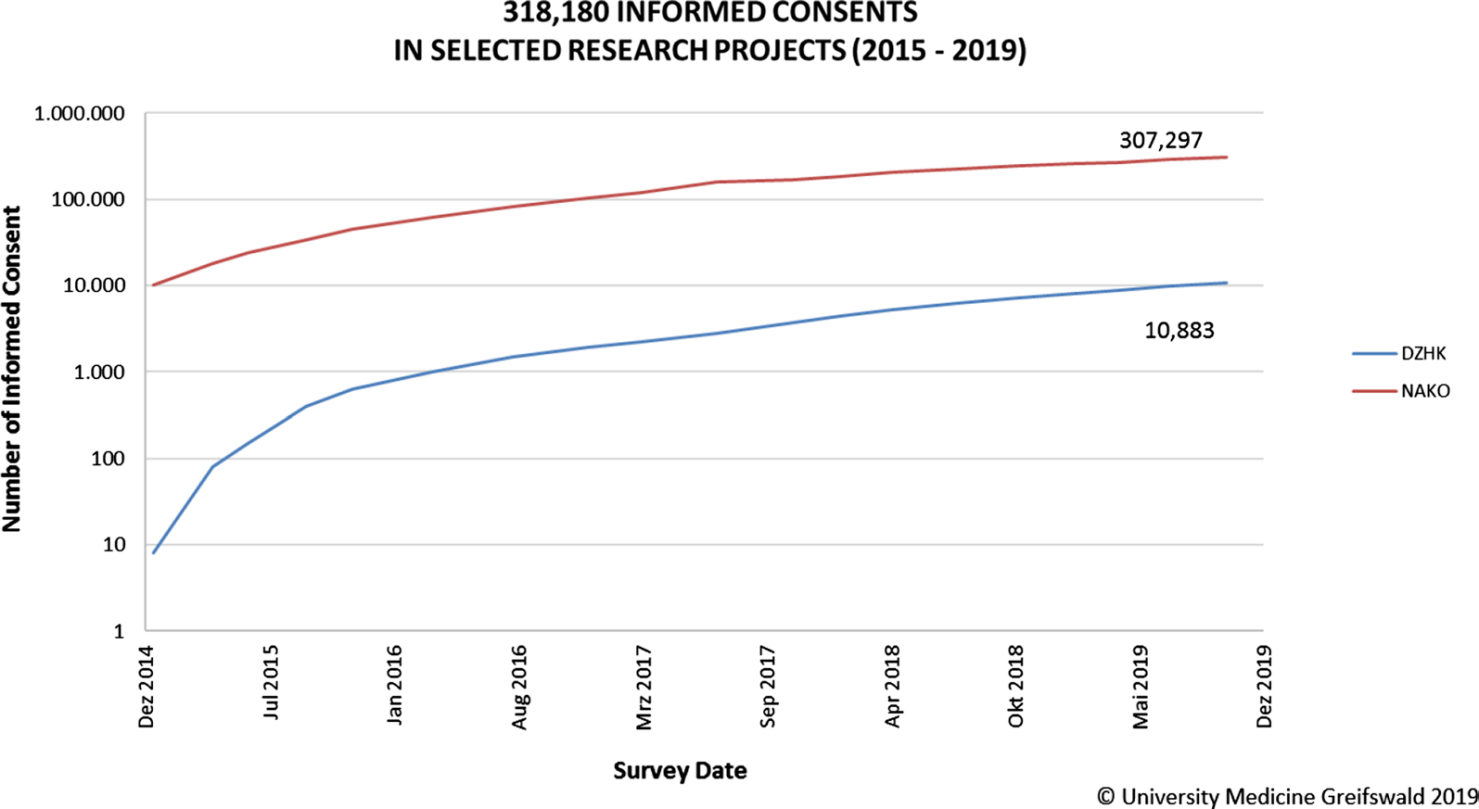
Summary of all managed consents within various research projects using gICS (as of October 2019; statistics for GANI_MED over time are not available—number of consents at project end: 13,934)

So far, numerous research projects were supported by gICS including large ones like the German National Cohort (NAKO, [9]), the German Centre for Cardiovascular Research (DZHK, [23]) and GANI_MED [24]. As a result, over 331 000 informed consents and over 2400 withdrawals were documented in these three research projects up until October 2019 (see Fig. 4).

### Benefits for research

#### Partial withdrawals and consent states

Since the modular approach of gICS provides a high level of granularity, partial withdrawals of single modules and even single policies are possible. As exemplarily shown for the NAKO project in Bialke et al. [25] research studies benefit from partial compared to full withdrawals. By providing the participant with the possibility of withdrawing his/her consent partially, analyses based on other policies can still be conducted. Consequently, the already gathered data can at least to some parts still be used for research—according to the not withdrawn consent policies, to which the participant had consented earlier. Therefore, this approach minimises the major risk in health care research regarding the unavailability of data.

**Fig. 5:**
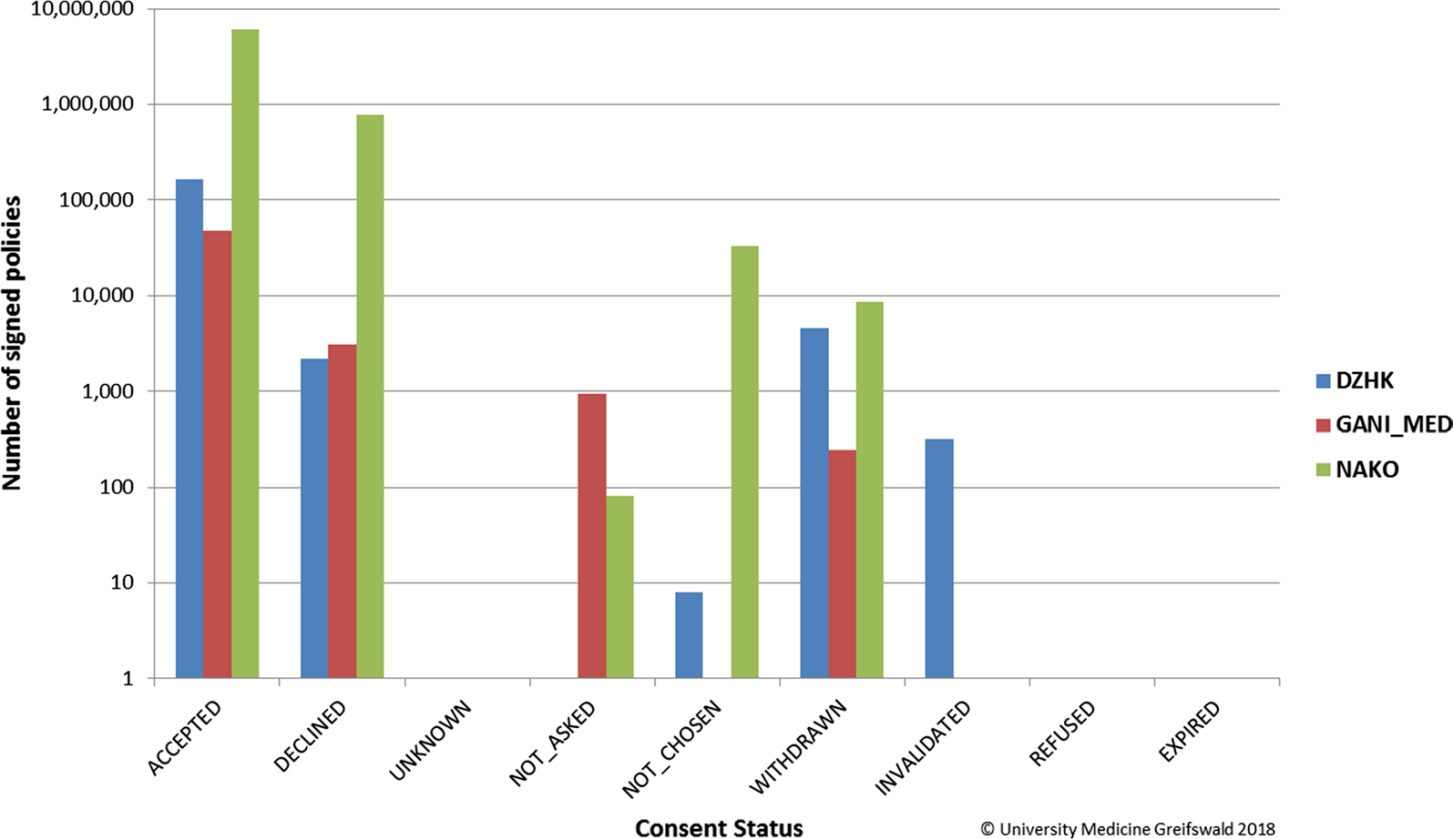
Overview of available consent status values within gICS (v.2.9.1) and their usage in the NAKO, DZHK and GANI_MED projects (logarithmic representation of the y-axis) as of October 2018

Using gICS also facilitates checks of consent states in real-time (see Fig. 5). This is especially important for data capture and usage in fast-paced medical environments with distributed infrastructure and various interfacing systems (e. g. Trusted Third Party, laboratory information or medical imaging management systems) as well as in projects with a very large amount of data, which make manual checks impractical.

#### Templates and versioning

Since research projects are very diverse, consents need to be adapted to the individual context. This can lead to time-consuming and costly developments in early study phases. Using templates for consents and versioning them as supported by gICS simplifies processes and can reduce the burden on study staff [25].

Furthermore, policies and consent templates tend to be added, edited or even deleted during the duration of long-term studies, e. g. the NAKO project. By managing different consent versions as supported by the gICS’ versioning, the burden on study staff as well as the central data management is further reduced.

Additionally, IC versioning with gICS adds the benefit of tracking changes over time, e. g. through amendments or new research modules. This feature is often needed in international research projects, which need support in providing consent forms in country-specific languages and/or with local adaptations.

#### Support of various clinical workflows

To support various workflows within a research study, different ways of collecting ICs with gICS are possible: 1) the electronic depiction of a paper-based consent and 2) fully electronic consent using the web-based service architecture of gICS.

The first workflow is mainly used in scenarios with unplanned visits such as registries. It includes the paper-based collection of consent, which is then scanned and stored in a digital format as a participant’s consent within gICS. A subsequent and, thus, asynchronous upload of the consent scan is possible.

The second workflow as in the NAKO project [9] uses SignPads for obtaining and recording digital signatures with biometric values (e. g. pressure, speed and fluency of writing movement) allowing fully electronic consent mechanisms. Thus, allowing the electronic consent to be gathered on-site (e. g. at the time of doctor’s consultation or participant’s physical stay at a study site). This approach is used within scenarios with planned visits, as in prospective cohort studies with regular follow-ups, to provide the necessary standardised hardware at every study site.

### Fulfilled requirements and use cases supported by gICS

As described, gICS creates and digitally manages modular ICs as well as withdrawals, and allows policy-specific queries. Table 2 depicts the existing functionalities according to the previously created requirements list (see Table 1).

## Discussion

Most tools for research are in-house or specifically tailored solutions with the disadvantages of varying technical staff or the end of tool support after a project’s finalisation. To ensure reusability in new projects as well as a widespread provision of a consent management tool for the scientific community, gICS is provided as open-source, free of charge software tool. Due to Docker, gICS is easy to install and supports research projects with limited resources regarding IT experts and finances. Additionally, gICS is suited for research projects with short as well as long project durations and has no practical limits regarding number of consents or consent template versions. Its generic approach and web-based client application allows necessary customisation—even in small and researcher-driven studies and registries with only a minimum of IT resources.

gICS supports all requirements and use cases regarding IC management as listed in Table 2. Requirements regarding IC structure and granularity as stated by the TMF [16] and GDPR [7] can be implemented and utilised using gICS. However, using gICS does not automatically guarantee a high degree of legal compliance, since the consent’s content is responsible for securing participants’ rights. gICS only supports the management of consent documents as well as permissions and prohibitions (regarding policies) stated by the participant. Using an IC is the responsibility of data management units and study sites [25]. The content of the informed consent and the resulting policies and modules have to be developed by the researcher—support, guidelines and requirements are provided in the literature [29] or at websites from institutions working in data protection. For example, text snippets and content templates for developing consent documents to use for research and to guarantee a high degree of legal compliance (in Germany) can be found at TMF [10]. gICS cannot substitute for an examination of the consent documents by the responsible ethics committee.

**Table 2:** List of requirements for a comprehensive informed consent management (see Table 1) and respective solutions using gICS

No.	Requirement and/or use case	gICS solution
1	Support of a general consent form for a research project	By design gICS facilitates to create general as well as project-specific templates for informed consents [25]
2	Support of individual participant consents	To address individual requirements of cohorts or groups of participants, the structure of each template can easily be adopted to the target groups’ needs using gICS
3	Clarity and transparency regarding each consent status to support Use and Access processes in compliance with data protection regulations	gICS supports depicting the participants’ will for each application scenario, such as Use and Access, in currently nine values: Accepted, Declined, Unknown, Not asked, Not chosen, Withdrawn, Invalidated, Refused and Expired [26]
4	Editing and updating a consent	The participant is enabled to change his/her will at any time. A given consent can be updated as well as withdrawn without any restrictions using gICS. For a precise chronological documentation every change results in a new “latest” and versioned consent easily manageable with gICS [25]
5	Support of consent exclusions	gICS supports the “opt-in” approach, which is the EU-GDPR default. To exclude specific purposes of data usage, the project consent has to contain a respective consent module, which can be accepted or declined by the participant. [25]
6	Possibility to define any number of (external) properties	To achieve a maximum of flexibility, the gICS data model allows to define gICS-specific properties (e. g. mandatory scans, scan size limits, permanent withdrawals, version and date weighting) and application-specific (external) properties (e. g. VALIDITY_PERIOD = p1y30d) for policies, modules and templates. [25, 27]
7	Possibility to define free text fields	Each consent template in gICS can be enhanced with free text fields of the types DATE, BOOLEAN, STRING, INTEGER or DOUBLE, e. g. to additionally document the name of a treatment facility. [25]
8	Possibility to withdraw consent (fully or partially)	The modular structure of consents within gICS facilitates partial and full withdrawals, e. g. as successfully applied in the German National Cohort. [25]
9	Support of consent versioning	Each policy, module and template of a consent references a specific version in gICS to ensure reproducibility. [25]
10	Possibility to freely configure automatable queries for consent status	gICS facilitates the functionality to request consent status (policy-based) via interface including further possible configurations as “ignoreVersionNumber” or unknownStateIsConsideredAsDecline [27, 28]
11	Possibility to define policies and combine them into modules to support fine-granular depiction of the participant’s expressed consent	gICS uses policies and modules as fine-granular as needed for a comprehensive consent representation [15].
12	Possibility to define mandatory policies/modules	Each consent module references assigned consent policies in gICS. For each consent template within gICS mandatory consent modules can easily be specified [25]
13	Possibility of automated search or query of individual, consented consent form, policies, modules or specific identifiers, e. g. case number	Using automated searches or queries of consent, policies, modules or specific identifiers already provided by gICS or manually created by the researcher, e. g. by using the search-function of the web frontend [25]
14	Support of exporting consented cases, e. g. by providing a list of participants’ pseudonyms with valid consents	gICS offers more than 50 comfort functionalities via a web service-based interface (SOAP) to manage and query consents and respective information. A complete list of functions and required parameters is provided in the online specification [25, 28]
15	Integration of paper-based workflows, e. g. attaching documents to a participant’s digital consent	Digitising and managing consents in gICS includes the possibility to upload one or more files (PDF-format, jpg), e. g. a scanned document of the participant’s paper-based consent, to the digital consent [25]
16	Management of domains (e. g. projects, study countries or sites)	Possibility to use specific domains in gICS and the gICS web frontend to manage those domains [25]
17	Intuitive usage and support of use cases	The up-to-date web-frontend of gICS supports the most common application scenarios while additional comfort functionalities, provided by the web-based interface, facilitate system integration purposes
18	Possibility to define the time of validity of a consent	Within gICS a consent’s time period of validity can be defined either by a fixed date, e. g. end of the research project, or by a dynamic date, e. g. 18th birthday of a study participant. This can be defined for domains, e.g. a study, specific consent templates, or modules

The status variables included in gICS are not restricted to the array of values used in the existing projects. Rather, they include values, which can be further adapted for future research projects. Nevertheless, certain preferences in the usage of those values can already be identified leading to the question of practical implications of the not-yet-used values. This will be evaluated with an interdisciplinary team and based on more research projects in the future. In any case, using a fine-granular consent with different consent status values, which can be directly exchanged between systems, allows the automated processing of granting or denying access to specific health-related information. Thus, gICS may also be suitable for the integration into an EHR system. However, gICS as an IC management tool does not provide authentication mechanisms. Such processes as well as implementations to handle and monitor authorisations have to be defined outside of the gICS-application, e. g. using a dispatcher.

According to MITRE [6], the future is to collect consents digitally. Until today, however, it is required to hand out a paper copy of the patient information as well as the signed consent form to study participants and patients in Germany. Consequently, this leads to a still paper-based process: Usually, when using SignPads to digitally capture consents the completed e-consent is printed out as a copy for the participant. However, a future gICS-feature could also include providing the consent electronically to the participant, e.g. via e-mail.

## Conclusions

The introduced approach of the generic Informed Consent Service gICS supports the automated processing of ICs, and use cases of a broad range of research projects to collect and manage ICs in compliance with their workflows as well as legal and ethical requirements.

gICS is under on-going further development and integration in work processes. For example, within the MAGIC-project (2016–2018) funded by the German Research Foundation (DFG) (grant number HO 1937/5-1) an FHIR-based exchange format was proposed [25]. Additionally, it will be used and further enhanced in the complex multi-site network MIRACUM (Medical Informatics in Research and Care in University Medicine; grant number: FKZ 01ZZ1801M) [30] leading most likely to further use cases.

A tool like gICS to simplify and support a sustainable IC management is a major key to successful study implementation and trust building with participants and the public. Therefore, interested researchers are invited to use gICS [21] and provide feedback for further improvements.

## Data Availability

gICS is available as download via TMF Toolpool [22], GitHub [21], the official website of the Trusted Third Party of the University Medicine Greifswald [20] and from the corresponding author on reasonable request.
